# Supplementary material for: Re-Irradiation by Stereotactic Radiotherapy of Brain Metastases in the Case of Local Recurrence
Source: Cancers (Basel). 2023 Feb 3;15(3):996. doi: 10.3390/cancers15030996 (PMC9913463; doi:10.3390/cancers15030996)
Supplement: Supplementary file 1 [file cancers-15-00996-s001.zip › cancers-2185495-supplementary.pdf]

Supplementary Materials: Reirradiation by Stereotactic Radiotherapy of Brain Metastases in Case of Local Recurrence

Ruben Touati, Vincent Bourbonne, Gurvan Dissaux, Gaëlle Goasduff, Olivier Pradier, Charles Peltier, Romuald Seizeur, Ulrike Schick and François Lucia

Table S1. Dosimetric parameters of first course of SRT.

**Dosimetric Parameters**	**34 metastases (*n* = 34) Median (Range) %**
GTV volume	1.8 (0.1–25.0)
PTV volume median	4.2 (0.7–37.2)
D_{min} GTV	25.82 Gy (21.09–29.98)
D_{max} GTV	32.83 (22.29–33.92)
D_{min} PTV	21.45 (19.79–28.68)
D_{max} PTV	32.83 (22.29–33.92)
Prescription isodose line	80.63% (71.26–96.52)
Target coverage GTV	100 (100–100)
Target coverage PTV	98.89 (86.19–99.76)
Delivered dose GTV	23.1 (21–23.1)
Delivered dose GTV BED	40.89 (35.7–40.89)
Delivered dose PTV	22.82 (19.91–23.04)
Delivered dose PTV BED	40.40 (35.24–40.79)
V_5 brain	5.31 (2.56–24.77)
V_{10} brain	1.85 (0.55–6.98)
V_{15} brain	0.82 (0.22–2.73)
V_{20} brain	0.36 (0.07–1.47)
V_{25} brain	0.07 (0–0.57)

Abbreviations: GTV = Gross Tumor Volume, PTV = Planning Target Volume, BED = Biologically Effective Dose, $V_{xGy\ (brain)}$ = volume of brain receiving dose larger than or equal to xGy, SRT = Stereotactic Radiation Therapy.

Table S2. Cumulative dosimetric parameters.

**Cumulative Dosimetric Parameters**	**34 metastases (*n* = 34) Median (Range) %**
D_{max} GTV	65.43 (45.01–75.51)
D_{max} PTV	65.43 (45.01–75.51)
Delivered dose GTV	46.20 (44.10–62.90)
Delivered dose GTV BED	81.78 (76.59–91.25)
Delivered dose PTV	45.90 (42.90–60.53)
Delivered dose PTV BED	81.24 (75.00–88.58)
V_5 brain	17.65 (6.91–101.04)
V_{10} brain	6.09 (2.59–59.83)
V_{15} brain	2.72 (1.07–34.43)
V_{20} brain	1.51 (0.47–17.84)
V_{25} brain	0.48 (0–8.85)

Table S3. Univariate and multivariate analysis for overall survival (OS).

Variables	Univariate Analysis			Multivariate Analysis		
	**HR**	**95% CI**	***p***	**HR**	**95% CI**	***p***
Histology (lung vs. others)	3.20	1.08–9.47	0.029	1.08	0.28–4.17	0.92
Age	1.01	0.96–1.07	0.60	-	-	-
Gender (Female vs. Male)	0.79	0.29–2.15	0.64	-	-	-
DS-GPA	1.16	0.64–2.11	0.63	-	-	-
RPA	1.30	0.54–3.11	0.56	-	-	-
Number of BM	0.66	0.34–1.30	0.12	-	-	-
ECM (yes vs. no)	1.29	0.64–2.63	0.47	-	-	-
KPS	0.013	0.0002–0.98	0.05	0.015	0.0002–1.18	0.06
Systemic treatment concomitant	0.66	0.25–1.73	0.40	-	-	-
GTV volume SRT1	1.03	0.95–1.12	0.54	-	-	-
PTV volume SRT1	1.01	0.95–1.06	0.81	-	-	-
GTV volume SRT2	1.00	0.97–1.03	0.98	-	-	-
PTV volume SRT2	1.00	0.98–1.02	0.97	-	-	-
Interval treatment time	1.04	0.99–1.09	0.12	-	-	-
Brain surgery SRT2 (yes vs. no)	10.18	1.33–78.12	0.006	6.84	1.06–61.02	0.04
Delivered dose GTV SRT1	0.47	0.17–1.32	0.23	-	-	-
Delivered dose GTV SRT1 BED	0.74	0.49–1.12	0.23	-	-	-
Delivered dose PTV SRT1	0.80	0.47–1.34	0.42	-	-	-
Delivered dose PTV SRT1 BED	0.87	0.66–1.15	0.35	-	-	-
Delivered dose GTV SRT2	0.96	0.87–1.07	0.43	-	-	-
Delivered dose GTV SRT2 BED	1.00	0.91–1.09	0.97	-	-	-
Delivered dose PTV SRT2	0.97	0.88–1.08	0.60	-	-	-
Delivered dose PTV SRT2 BED	1.02	0.93–1.12	0.70	-	-	-
Delivered dose GTV total	0.92	0.81–1.05	0.17	-	-	-
Delivered dose GTV total BED	0.95	0.85–1.06	0.35	-	-	-
Delivered dose PTV total	0.93	0.83–1.05	0.21	-	-	-
Delivered dose PTV total BED	0.96	0.87–1.07	0.46	-	-	-
Dmax SRT1	1.00	0.89–1.14	0.95	-	-	-
Dmax SRT2	0.97	0.87–1.09	0.60	-	-	-
Dmax total	1.00	0.94–1.06	0.93	-	-	-
V_5 brain SRT1	0.99	0.91–1.07	0.75			
V_5 brain SRT2	0.98	0.94–1.02	0.15			
V_{10} brain SRT1	0.99	0.77–1.28	0.96			
V_{10} brain SRT2	0.96	0.89–1.04	0.17			
V_{15} brain SRT1	0.88	0.46–1.69	0.71			
V_{15} brain SRT2	0.92	0.79–1.07	0.13			
V_{20} brain SRT1	0.60	0.16–2.34	0.44			
V_{20} brain SRT2	0.84	0.62–1.13	0.10			
V_{25} brain SRT1	0.16	0.005–5.12	0.25			
V_{25} brain SRT2	0.64	0.33–1.23	0.05	0.74	0.41–1.34	0.18

Abbreviations: HR = hazard ratio, CI = confidence interval, DS-GPA = Diagnostic-Specific Graded Prognostic Assessment, RPA = Recursive partitioning analysis, BM = Brain Metastases, ECM = Extra cranial metastases, KPS = Karnofsky Performance Status, SRT1 = first course of Stereotactic Radiation Therapy, SRT2 = second course of Stereotactic Radiation Therapy.
